# *N*-[(2*S*)-2-(4-Bromo­phen­yl)-4-oxo-1,3-thia­zolidin-3-yl]pyridine-3-carboxamide

**DOI:** 10.1107/S1600536810022506

**Published:** 2010-06-18

**Authors:** Mehmet Akkurt, Ísmail Çelik, Hale Demir, Sumru Özkırımlı, Orhan Büyükgüngör

**Affiliations:** a Department of Physics, Faculty of Arts and Sciences, Erciyes University, 38039 Kayseri, Turkey; b Department of Physics, Faculty of Arts and Sciences, Cumhuriyet University, 58140 Sivas, Turkey; c Department of Pharmaceutical Chemistry, Faculty of Pharmacy, Istanbul University, 34116 Beyazit, Istanbul, Turkey; d Department of Physics, Faculty of Arts and Sciences, Ondokuz Mayıs University, 55139 Samsun, Turkey

## Abstract

In the title compound, C_15_H_12_BrN_3_O_2_S, the dihedral angle between the pyridine and benzene rings is 73.17 (19)°. The five-membered 1,3-thia­zolidine ring has an envelope conformation, with the S atom displaced by 0.196 (1) Å from the mean plane of the four other ring atoms. An intra­molecular C—H⋯N inter­action occurs. The crystal structure is stabil­ized by inter­molecular N—H⋯O and C—H⋯O hydrogen bonds and C—H⋯π inter­actions. In addition, a weak π–π stacking inter­action is also observed between the 1,3-thia­zolidine and pyridine rings [centroid–centroid distance = 3.805 (2) Å].

## Related literature

For the cytoprotective and anti­viral properties of nicotinamide, see: Gaudineau & Auclair (2004 ▶); Moell *et al.* (2009 ▶). For 3-pyridine­carboxamide derivatives with anti­tumor activity, see: Elbaum *et al.* (2003 ▶). For the various biological activities of thia­zolidinones, see: Capan *et al.* (1999 ▶) and Ozkırımlı *et al.* (2009 ▶) (anti­fungal); Guzel *et al.* (2006 ▶) (anti­tuberculosis); Rawal *et al.* (2007 ▶) (RT Inhibitor); Vanderlinden *et al.* (2010 ▶) (anti­viral). For standard bond-length data, see: Allen *et al.* (1987 ▶). For puckering and asymmetry parameters, see: Cremer & Pople (1975 ▶).
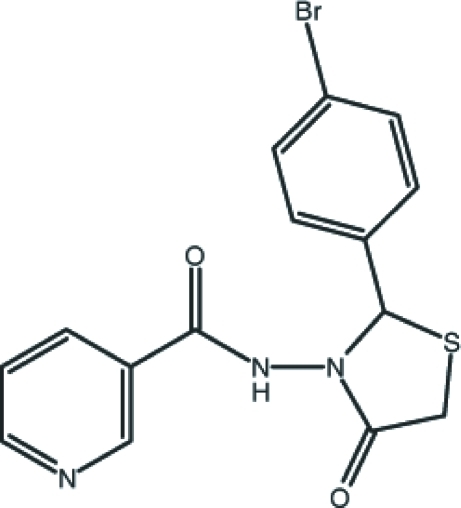


## Experimental

### 

#### Crystal data

C_15_H_12_BrN_3_O_2_S
*M* *_r_* = 378.25
Trigonal, 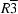

*a* = 24.9588 (9) Å
*c* = 12.8013 (5) Å
*V* = 6906.1 (4) Å^3^
*Z* = 18
Mo *K*α radiation
μ = 2.82 mm^−1^
*T* = 296 K
0.28 × 0.23 × 0.19 mm

#### Data collection

Stoe IPDS 2 diffractometer
Absorption correction: integration (*X-RED32*; Stoe & Cie, 2002 ▶) *T*_min_ = 0.505, *T*_max_ = 0.616
13554 measured reflections
3174 independent reflections
1963 reflections with *I* > 2σ(*I*)
*R*_int_ = 0.050

#### Refinement

*R*[*F* ^2^ > 2σ(*F* ^2^)] = 0.042
*wR*(*F* ^2^) = 0.085
*S* = 0.95
3174 reflections
202 parameters
1 restraint
H atoms treated by a mixture of independent and constrained refinement
Δρ_max_ = 0.22 e Å^−3^
Δρ_min_ = −0.35 e Å^−3^

### 

Data collection: *X-AREA* (Stoe & Cie, 2002 ▶); cell refinement: *X-AREA*; data reduction: *X-RED32* (Stoe & Cie, 2002 ▶); program(s) used to solve structure: *SIR97* (Altomare *et al.*, 1999 ▶); program(s) used to refine structure: *SHELXL97* (Sheldrick, 2008 ▶); molecular graphics: *ORTEP-3* (Farrugia, 1997 ▶); software used to prepare material for publication: *WinGX* (Farrugia, 1999 ▶).

## Supplementary Material

Crystal structure: contains datablocks global, I. DOI: 10.1107/S1600536810022506/lh5067sup1.cif

Structure factors: contains datablocks I. DOI: 10.1107/S1600536810022506/lh5067Isup2.hkl

Additional supplementary materials:  crystallographic information; 3D view; checkCIF report

## Figures and Tables

**Table 1:** Hydrogen-bond geometry (Å, °) *Cg*3 is the centroid of the C1–C6 benzene ring.

*D*—H⋯*A*	*D*—H	H⋯*A*	*D*⋯*A*	*D*—H⋯*A*
N2—H2*A*⋯O1^i^	0.85 (3)	2.07 (3)	2.914 (4)	172 (4)
C3—H3⋯O2^i^	0.93	2.43	3.237 (5)	146
C15—H15⋯N2	0.93	2.54	2.864 (5)	101
C15—H15⋯O1^i^	0.93	2.50	3.399 (5)	162
C14—H14⋯*Cg*3^ii^	0.93	2.79	3.692 (4)	164

Symmetry codes: (i) 
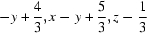
; (ii) 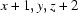.

**Fig. 1.**
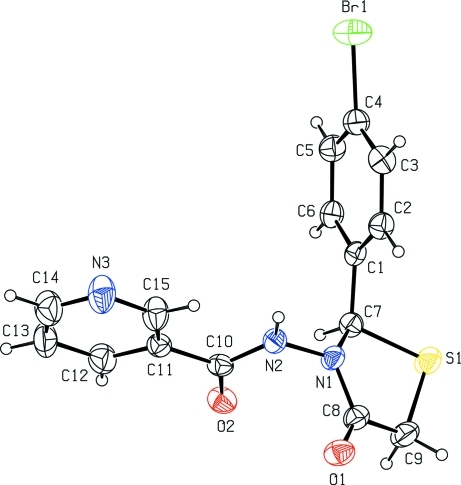
The title molecule with the atom numbering scheme. Displacement ellipsoids for non-H atoms are drawn at the 30% probability level.

**Fig. 2.**
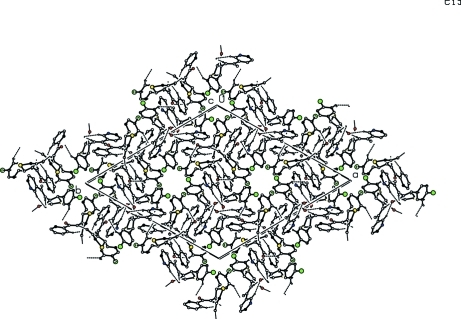
View of the packing and hydrogen bonding interactions of (I). All hydrogen atoms not involved in hydrogen bonding have been omitted for clarity.

## supplementary crystallographic information

### Comment

Development of new active compounds for viral infections is a high priority goal. The rapid onset of resistance and hypersensitivity reactions limit the use of antiviral compounds and therefore, there is an ongoing need for novel antiviral agents. A number of diverse chemical structures have been shown to be potent RT Inhibitors. Nicotinamide is gaining attention for its cytoprotective and antiviral properties (Gaudineau *et al.*, 2004). Antiviral effect of nicotinamide and its inhibitory effect on enterovirus induced chemokine secretion have been recently shown (Moell *et al.*, 2009). Furthermore, 3-pyridinecarboxamide derivatives with antitumor activity have been reported (Elbaum *et al.*, 2003). Thiazolidinones exhibit various biological activities such as antifungal (Capan *et al.*, 1999; Ozkırımlı *et al.*, 2009); antituberculosis (Guzel *et al.*, 2006); RT Inhibitor (Rawal *et al.*, 2007); antiviral (Vanderlinden *et al.*, 2010). We combine these two moities as part of an ongoing project directed towards the design and synthesis of bioactive molecules bearing 4-thiazolidinone and pyridine-3-carboxamide scaffolds together.

In the title molecule (I) shown in Fig. 1, the bond lengths and the bond angles are in the normal ranges (Allen *et al.*, 1987). The C2—C1—C7—N1, C2—C1—C7—S1, N1—N2—C10—O2 and N1—N2—C10—C11 torsion angles are 40.2 (4), -76.2 (3), -0.7 (5) and -179.7 (3) °, respectively. The dihedral angle between the pyridine (N3/C11–C15) and benzene (C1–C6) rings is 73.17 (19) °. The five-membered 1,3-thiazolidine ring has an envelope conformation, with atom S1 displaced by -0.196 (1) Å from the S1/N1/C7–C9 plane [the puckering parameters (Cremer & Pople, 1975) are Q_2_ = 0.361 (3) Å and φ_2_ = 188.0 (5) °].

The crystal structure is stabilized by intermolecular N—H···O and C—H···O hydrogen bonding interactions (Table 1, Fig. 2) and a C—H···π interactions (Table 1). A weak π-π stacking interaction is observed between the 1,3-thiazolidine and pyridine rings [*Cg*2···*Cg*2(2/3 - *x*, 7/3 - *y*, 1/3 - *z*) = 3.805 (2) Å, where *Cg*1 and *Cg*2 are the centroids of the S1/N1/C7–C9 1,3-thiazolidine and N3/C11–C15 pyridine rings, respectively].

### Experimental

0.01 mol of *N*'-(4-bromobenzylidine)pyridine-3-carbohydrazide was reacted with 0.03 mol of mercaptoacetic acid in anhydrous benzene for 8 h using a Dean-Stark trap. Excess benzene was removed under reduced pressure. The residue was triturated with saturated sodium bicarbonate solution. The separated solid was filtered, washed with water and crystallized from methanol. White crystalline solid. Yield: 60.84%; m.p.: 446.1–450.0 K. UV (EtOH) max: 202.6, 221.2, 264.8 nm. IR (KBr) υ: 1666 (amide C=O), 1687 (thia C=O); ^1^H-NMR (DMSO-d_6_, 400 MHz): 3.80 (1*H*, d, J=16 Hz, H5-thia.), 3.95 (1*H*, dd, J=15.8, 2.8 Hz, H5-thia.), 5.92 (1*H*, s, H2-thia.), 7.46 (2*H*, d, J=8.4 Hz, 2-C_6_H_4_-(H2,6)-thia.), 7.47–7.49 (1*H*, m, H5-pyridine), 7.56 (1*H*, d, J=8.8 Hz, 2-C_6_H_4_-(H3,5)-thia.), 8.08 (1*H*, dt, J=8.4, 1.6, 1.6 Hz, H4-pyridine), 8.71 (1*H*, dd, J=4.6, 1.6 Hz, H6-pyridine), 8.87 (1*H*, d, J=1.6 Hz, H2-pyridine), 10.93 (1*H*, s, CONH); ESI+ (m/*z*): 380.23 ([MH+2]^+^, 100), 378.24([MH]^+^, 98.71). Analysis calculated for C_15_H_12_BrN_3_O_2_S: C 47.63, H 3.20, N 11.11%. Found: C 47.39, H 3.09, N 10.98%.

### Refinement

The C-bound H atoms were geometrically placed (C—H = 0.93–0.97 Å) and refined as riding with *U*_iso_(H) = 1.2*U*_eq_(C). The N-bound H atoms were located from the Fourier synthesis and restrained to 0.86 (2) Å, and refined with *U*_iso_(H) = 1.5*U*_eq_(N).

### Crystal data

**Table:**

C_15_H_12_BrN_3_O_2_S	*D*_x_ = 1.637 Mg m^−^^3^
*M**_r_* = 378.25	Mo *K*α radiation, λ = 0.71073 Å
Trigonal, *R*3	Cell parameters from 10912 reflections
Hall symbol: -R 3	θ = 1.6–28.0°
*a* = 24.9588 (9) Å	µ = 2.82 mm^−^^1^
*c* = 12.8013 (5) Å	*T* = 296 K
*V* = 6906.1 (4) Å^3^	Block, colourless
*Z* = 18	0.28 × 0.23 × 0.19 mm
*F*(000) = 3420	

### Data collection

**Table:**

Stoe IPDS 2 diffractometer	3174 independent reflections
Radiation source: sealed X-ray tube, 12 x 0.4 mm long-fine focus	1963 reflections with *I* > 2σ(*I*)
plane graphite	*R*_int_ = 0.050
Detector resolution: 6.67 pixels mm^-1^	θ_max_ = 26.5°, θ_min_ = 1.6°
ω scans	*h* = −30→27
Absorption correction: integration (*X-RED32*; Stoe & Cie, 2002)	*k* = −31→31
*T*_min_ = 0.505, *T*_max_ = 0.616	*l* = −15→16
13554 measured reflections	

### Refinement

**Table:**

Refinement on *F*^2^	Primary atom site location: structure-invariant direct methods
Least-squares matrix: full	Secondary atom site location: difference Fourier map
*R*[*F*^2^ > 2σ(*F*^2^)] = 0.042	Hydrogen site location: inferred from neighbouring sites
*wR*(*F*^2^) = 0.085	H atoms treated by a mixture of independent and constrained refinement
*S* = 0.95	*w* = 1/[σ^2^(*F*_o_^2^) + (0.0375*P*)^2^] where *P* = (*F*_o_^2^ + 2*F*_c_^2^)/3
3174 reflections	(Δ/σ)_max_ < 0.001
202 parameters	Δρ_max_ = 0.22 e Å^−^^3^
1 restraint	Δρ_min_ = −0.35 e Å^−^^3^

### Special details

**Table:**

Geometry. Bond distances, angles *etc*. have been calculated using the rounded fractional coordinates. All su's are estimated from the variances of the (full) variance-covariance matrix. The cell e.s.d.'s are taken into account in the estimation of distances, angles and torsion angles
Refinement. Refinement on *F*^2^ for ALL reflections except those flagged by the user for potential systematic errors. Weighted *R*-factors *wR* and all goodnesses of fit *S* are based on *F*^2^, conventional *R*-factors *R* are based on *F*, with *F* set to zero for negative *F*^2^. The observed criterion of *F*^2^ > σ(*F*^2^) is used only for calculating -*R*-factor-obs *etc*. and is not relevant to the choice of reflections for refinement. *R*-factors based on *F*^2^ are statistically about twice as large as those based on *F*, and *R*-factors based on ALL data will be even larger.

### Fractional atomic coordinates and isotropic or equivalent isotropic displacement parameters (Å^2^)

**Table:**

	*x*	*y*	*z*	*U*_iso_*/*U*_eq_	
Br1	0.04324 (2)	0.93003 (2)	0.13333 (3)	0.0833 (2)	
S1	0.18291 (4)	0.97254 (4)	0.63617 (6)	0.0594 (3)	
O1	0.35556 (11)	1.03443 (11)	0.58153 (16)	0.0609 (9)	
O2	0.33205 (10)	1.15286 (10)	0.51669 (15)	0.0561 (8)	
N1	0.27161 (11)	1.03012 (11)	0.50268 (17)	0.0459 (8)	
N2	0.30533 (11)	1.06767 (12)	0.41953 (17)	0.0456 (8)	
N3	0.41259 (16)	1.17793 (16)	0.1703 (2)	0.0802 (13)	
C1	0.16764 (13)	0.99737 (13)	0.4306 (2)	0.0403 (9)	
C2	0.16483 (14)	0.95013 (14)	0.3696 (2)	0.0496 (11)	
C3	0.12676 (15)	0.92888 (15)	0.2825 (2)	0.0533 (11)	
C4	0.09162 (14)	0.95549 (15)	0.2568 (2)	0.0522 (11)	
C5	0.09214 (14)	1.00079 (15)	0.3177 (2)	0.0534 (11)	
C6	0.13042 (14)	1.02166 (14)	0.4045 (2)	0.0474 (10)	
C7	0.21047 (13)	1.02331 (14)	0.5225 (2)	0.0444 (10)	
C8	0.30194 (16)	1.02317 (14)	0.5850 (2)	0.0488 (11)	
C9	0.26115 (15)	1.00121 (17)	0.6800 (2)	0.0610 (11)	
C10	0.33513 (13)	1.13027 (15)	0.4341 (2)	0.0443 (10)	
C11	0.37035 (14)	1.16854 (14)	0.3424 (2)	0.0451 (10)	
C12	0.39270 (18)	1.23136 (16)	0.3450 (3)	0.0687 (14)	
C13	0.4255 (2)	1.26670 (18)	0.2588 (3)	0.0816 (16)	
C14	0.43328 (19)	1.2376 (2)	0.1757 (3)	0.0802 (17)	
C15	0.38243 (17)	1.14490 (17)	0.2539 (3)	0.0653 (14)	
H2	0.18870	0.93250	0.38740	0.0600*	
H2A	0.3055 (19)	1.0477 (17)	0.366 (2)	0.1000*	
H3	0.12500	0.89710	0.24190	0.0640*	
H5	0.06710	1.01740	0.30110	0.0640*	
H6	0.13100	1.05260	0.44590	0.0570*	
H7	0.21550	1.06370	0.54110	0.0530*	
H9A	0.27310	1.03500	0.72880	0.0730*	
H9B	0.26480	0.96860	0.71470	0.0730*	
H12	0.38610	1.24980	0.40290	0.0820*	
H13	0.44160	1.30930	0.25830	0.0980*	
H14	0.45490	1.26170	0.11850	0.0960*	
H15	0.36840	1.10270	0.25250	0.0780*	

### Atomic displacement parameters (Å^2^)

**Table:**

	*U*^11^	*U*^22^	*U*^33^	*U*^12^	*U*^13^	*U*^23^
Br1	0.0755 (3)	0.1114 (4)	0.0638 (2)	0.0473 (3)	−0.0204 (2)	−0.0167 (2)
S1	0.0544 (5)	0.0724 (6)	0.0463 (4)	0.0279 (5)	0.0077 (4)	0.0159 (4)
O1	0.0534 (15)	0.0774 (17)	0.0589 (13)	0.0380 (13)	−0.0050 (11)	0.0014 (11)
O2	0.0534 (14)	0.0621 (14)	0.0462 (12)	0.0240 (12)	0.0031 (9)	−0.0101 (10)
N1	0.0430 (14)	0.0531 (16)	0.0386 (12)	0.0218 (13)	0.0041 (11)	0.0096 (11)
N2	0.0462 (15)	0.0478 (16)	0.0370 (13)	0.0192 (13)	0.0057 (11)	0.0030 (11)
N3	0.095 (3)	0.068 (2)	0.0646 (18)	0.031 (2)	0.0335 (17)	0.0100 (16)
C1	0.0415 (17)	0.0418 (17)	0.0388 (14)	0.0217 (14)	0.0059 (12)	0.0077 (12)
C2	0.0474 (19)	0.052 (2)	0.0564 (17)	0.0300 (16)	0.0032 (14)	0.0058 (14)
C3	0.053 (2)	0.052 (2)	0.0570 (18)	0.0278 (17)	0.0042 (15)	−0.0056 (15)
C4	0.0463 (19)	0.058 (2)	0.0486 (16)	0.0233 (17)	0.0024 (14)	0.0021 (15)
C5	0.0477 (19)	0.056 (2)	0.0630 (19)	0.0309 (17)	−0.0008 (15)	0.0061 (16)
C6	0.0514 (19)	0.0434 (18)	0.0514 (16)	0.0268 (16)	0.0050 (14)	0.0040 (13)
C7	0.0421 (18)	0.0465 (18)	0.0456 (15)	0.0229 (15)	0.0027 (13)	0.0031 (13)
C8	0.058 (2)	0.0515 (19)	0.0404 (16)	0.0299 (17)	−0.0024 (14)	−0.0005 (13)
C9	0.064 (2)	0.076 (2)	0.0430 (17)	0.035 (2)	−0.0016 (15)	0.0114 (16)
C10	0.0359 (16)	0.053 (2)	0.0425 (16)	0.0212 (15)	−0.0028 (12)	−0.0033 (14)
C11	0.0401 (17)	0.0482 (19)	0.0452 (16)	0.0207 (15)	−0.0005 (13)	0.0004 (13)
C12	0.080 (3)	0.056 (2)	0.058 (2)	0.025 (2)	0.0008 (18)	−0.0047 (17)
C13	0.098 (3)	0.044 (2)	0.078 (3)	0.017 (2)	0.006 (2)	0.0110 (19)
C14	0.078 (3)	0.071 (3)	0.070 (3)	0.021 (2)	0.021 (2)	0.011 (2)
C15	0.076 (3)	0.057 (2)	0.0571 (19)	0.029 (2)	0.0231 (17)	0.0053 (17)

### Geometric parameters (Å, °)

**Table:**

Br1—C4	1.896 (3)	C5—C6	1.386 (4)
S1—C7	1.823 (3)	C8—C9	1.503 (4)
S1—C9	1.801 (4)	C10—C11	1.491 (4)
O1—C8	1.223 (5)	C11—C12	1.377 (5)
O2—C10	1.219 (3)	C11—C15	1.379 (5)
N1—N2	1.390 (3)	C12—C13	1.394 (6)
N1—C7	1.471 (5)	C13—C14	1.356 (6)
N1—C8	1.358 (4)	C2—H2	0.9300
N2—C10	1.366 (4)	C3—H3	0.9300
N3—C14	1.312 (6)	C5—H5	0.9300
N3—C15	1.331 (5)	C6—H6	0.9300
N2—H2A	0.85 (3)	C7—H7	0.9800
C1—C6	1.380 (5)	C9—H9A	0.9700
C1—C7	1.501 (4)	C9—H9B	0.9700
C1—C2	1.386 (4)	C12—H12	0.9300
C2—C3	1.387 (4)	C13—H13	0.9300
C3—C4	1.379 (5)	C14—H14	0.9300
C4—C5	1.368 (4)	C15—H15	0.9300
			
Br1···H5^i^	3.1900	C15···O1^vi^	3.399 (5)
S1···C6^ii^	3.524 (4)	C1···H6^ii^	2.9800
S1···N3^iii^	3.003 (3)	C2···H7^ii^	2.7800
S1···C14^iii^	3.644 (5)	C3···H7^ii^	2.9100
S1···H6^ii^	3.0300	C4···H14^viii^	3.0000
O1···N2	2.757 (4)	C5···H5^i^	3.0700
O1···C10	3.281 (4)	C5···H14^viii^	2.9100
O1···N2^iv^	2.914 (4)	C6···H14^viii^	3.0100
O1···C2^iv^	3.369 (4)	C6···H6^ii^	2.9700
O1···C15^iv^	3.399 (6)	C8···H2A^iv^	3.00 (6)
O2···C2^v^	3.415 (6)	C10···H3^iv^	2.9500
O2···C8	3.062 (4)	C10···H7	2.9300
O2···N1	2.659 (3)	C12···H9B^v^	3.0200
O2···C7	3.140 (4)	C15···H2A	2.64 (4)
O2···C3^iv^	3.237 (6)	H2···N1	2.7100
O1···H15^iv^	2.5000	H2···O2^ii^	2.7500
O1···H2A^iv^	2.07 (5)	H2A···C15	2.64 (4)
O2···H7	2.6500	H2A···H15	2.0700
O2···H2^v^	2.7500	H2A···O1^vi^	2.07 (3)
O2···H12	2.5600	H2A···C8^vi^	3.00 (4)
O2···H3^iv^	2.4300	H3···O2^vi^	2.4300
N1···O2	2.659 (3)	H3···C10^vi^	2.9500
N2···O1	2.757 (4)	H5···Br1^ix^	3.1900
N2···C2	3.320 (4)	H5···C5^ix^	3.0700
N2···O1^vi^	2.914 (4)	H6···H7	2.3300
N3···C9^vii^	3.262 (5)	H6···S1^v^	3.0300
N3···S1^vii^	3.003 (3)	H6···C1^v^	2.9800
N1···H2	2.7100	H6···C6^v^	2.9700
N2···H15	2.5400	H7···O2	2.6500
N3···H9B^vii^	2.8500	H7···C10	2.9300
C2···N2	3.320 (4)	H7···H6	2.3300
C2···O2^ii^	3.415 (4)	H7···C2^v^	2.7800
C2···O1^vi^	3.369 (5)	H7···C3^v^	2.9100
C3···O2^vi^	3.237 (5)	H9B···C12^ii^	3.0200
C6···S1^v^	3.524 (3)	H9B···N3^iii^	2.8500
C7···O2	3.140 (4)	H12···O2	2.5600
C8···O2	3.062 (4)	H14···C4^viii^	3.0000
C9···N3^iii^	3.262 (5)	H14···C5^viii^	2.9100
C10···O1	3.281 (4)	H14···C6^viii^	3.0100
C14···S1^vii^	3.644 (4)	H15···N2	2.5400
C14···C15^viii^	3.507 (7)	H15···H2A	2.0700
C15···C14^viii^	3.507 (7)	H15···O1^vi^	2.5000
			
C7—S1—C9	90.87 (15)	C10—C11—C15	123.9 (3)
N2—N1—C7	117.0 (2)	C11—C12—C13	118.5 (3)
N2—N1—C8	119.5 (3)	C12—C13—C14	118.6 (4)
C7—N1—C8	117.6 (2)	N3—C14—C13	124.7 (4)
N1—N2—C10	117.8 (2)	N3—C15—C11	124.9 (3)
C14—N3—C15	116.1 (3)	C1—C2—H2	120.00
C10—N2—H2A	129 (2)	C3—C2—H2	120.00
N1—N2—H2A	114 (2)	C2—C3—H3	120.00
C2—C1—C7	122.0 (3)	C4—C3—H3	120.00
C2—C1—C6	118.6 (3)	C4—C5—H5	120.00
C6—C1—C7	119.4 (3)	C6—C5—H5	120.00
C1—C2—C3	120.8 (3)	C1—C6—H6	119.00
C2—C3—C4	119.1 (3)	C5—C6—H6	119.00
C3—C4—C5	121.1 (3)	S1—C7—H7	109.00
Br1—C4—C3	119.3 (2)	N1—C7—H7	109.00
Br1—C4—C5	119.6 (3)	C1—C7—H7	109.00
C4—C5—C6	119.2 (3)	S1—C9—H9A	110.00
C1—C6—C5	121.2 (3)	S1—C9—H9B	110.00
S1—C7—C1	112.7 (2)	C8—C9—H9A	110.00
S1—C7—N1	103.1 (2)	C8—C9—H9B	110.00
N1—C7—C1	112.9 (2)	H9A—C9—H9B	109.00
N1—C8—C9	110.8 (3)	C11—C12—H12	121.00
O1—C8—C9	125.3 (3)	C13—C12—H12	121.00
O1—C8—N1	123.9 (3)	C12—C13—H13	121.00
S1—C9—C8	107.2 (2)	C14—C13—H13	121.00
N2—C10—C11	115.8 (2)	N3—C14—H14	118.00
O2—C10—N2	121.6 (3)	C13—C14—H14	118.00
O2—C10—C11	122.7 (3)	N3—C15—H15	118.00
C12—C11—C15	117.2 (3)	C11—C15—H15	118.00
C10—C11—C12	118.9 (3)		
			
C7—S1—C9—C8	−26.5 (3)	C7—C1—C2—C3	−177.2 (3)
C9—S1—C7—N1	29.0 (2)	C6—C1—C7—S1	104.9 (3)
C9—S1—C7—C1	151.0 (3)	C6—C1—C7—N1	−138.8 (3)
C7—N1—N2—C10	78.1 (4)	C1—C2—C3—C4	0.2 (5)
C8—N1—C7—S1	−26.4 (3)	C2—C3—C4—C5	−2.3 (5)
N2—N1—C7—C1	58.8 (3)	C2—C3—C4—Br1	176.3 (2)
N2—N1—C7—S1	−179.29 (19)	Br1—C4—C5—C6	−176.3 (2)
N2—N1—C8—O1	−19.3 (4)	C3—C4—C5—C6	2.4 (5)
C7—N1—C8—O1	−171.5 (3)	C4—C5—C6—C1	−0.3 (5)
N2—N1—C8—C9	159.6 (3)	O1—C8—C9—S1	−165.2 (3)
C7—N1—C8—C9	7.4 (4)	N1—C8—C9—S1	16.0 (3)
C8—N1—C7—C1	−148.3 (3)	N2—C10—C11—C12	169.1 (4)
C8—N1—N2—C10	−74.3 (4)	O2—C10—C11—C12	−9.9 (6)
N1—N2—C10—O2	−0.7 (5)	O2—C10—C11—C15	169.9 (4)
N1—N2—C10—C11	−179.7 (3)	N2—C10—C11—C15	−11.1 (6)
C14—N3—C15—C11	1.9 (7)	C15—C11—C12—C13	0.3 (6)
C15—N3—C14—C13	−0.6 (8)	C10—C11—C15—N3	178.5 (4)
C6—C1—C2—C3	1.8 (5)	C10—C11—C12—C13	−179.9 (4)
C2—C1—C6—C5	−1.7 (5)	C12—C11—C15—N3	−1.8 (7)
C2—C1—C7—N1	40.2 (4)	C11—C12—C13—C14	0.8 (7)
C7—C1—C6—C5	177.3 (3)	C12—C13—C14—N3	−0.7 (8)
C2—C1—C7—S1	−76.2 (3)		

Symmetry codes: (i) −*x*+*y*−1, −*x*+1, *z*; (ii) *y*−1, −*x*+*y*, −*z*+1; (iii) −*y*+4/3, *x*−*y*+5/3, *z*+2/3; (iv) −*x*+*y*−1/3, −*x*+4/3, *z*+1/3; (v) *x*−*y*+1, *x*+1, −*z*+1; (vi) −*y*+4/3, *x*−*y*+5/3, *z*−1/3; (vii) −*x*+*y*−1/3, −*x*+4/3, *z*−2/3; (viii) −*x*+2/3, −*y*+7/3, −*z*+1/3; (ix) −*y*+1, *x*−*y*+2, *z*.

### Hydrogen-bond geometry (Å, °)

**Table:**

Cg3 is the centroid of the C1–C6 benzene ring.

**Table:**

*D*—H···*A*	*D*—H	H···*A*	*D*···*A*	*D*—H···*A*
N2—H2A···O1^vi^	0.85 (3)	2.07 (3)	2.914 (4)	172 (4)
C3—H3···O2^vi^	0.93	2.43	3.237 (5)	146
C15—H15···N2	0.93	2.54	2.864 (5)	101
C15—H15···O1^vi^	0.93	2.50	3.399 (5)	162
C14—H14···Cg3i	0.93	2.79	3.692 (4)	164

Symmetry codes: (vi) −*y*+4/3, *x*−*y*+5/3, *z*−1/3; i.
